# Impact of deprivation and rural residence on treatment of colorectal and lung cancer

**DOI:** 10.1038/sj.bjc.6600515

**Published:** 2002-09-09

**Authors:** N C Campbell, A M Elliott, L Sharp, L D Ritchie, J Cassidy, J Little

**Affiliations:** Department of General Practice and Primary Care, Foresterhill Health Centre, Westburn Road, Aberdeen AB25 2AY, UK; Department of Medicine and Therapeutics, Aberdeen University Medical School, Foresterhill, Aberdeen AB25 2ZD, UK

**Keywords:** Lung neoplasms, colorectal neoplasms, delivery of healthcare, socioeconomic factors rural population urban population

## Abstract

For common cancers, survival is poorer for deprived and outlying, rural patients. This study investigated whether there were differences in treatment of colorectal and lung cancer in these groups. Case notes of 1314 patients in north and northeast Scotland who were diagnosed with lung or colorectal cancer in 1995 or 1996 were reviewed. On univariate analysis, the proportions of patients receiving surgery, chemotherapy and radiotherapy appeared similar in all socio-economic and rural categories. Adjusting for disease stage, age and other factors, there was less chemotherapy among deprived patients with lung cancer (odds ratio 0.39; 95% confidence intervals 0.16 to 0.96) and less radiotherapy among outlying patients with colorectal cancer (0.39; 0.19 to 0.82). The time between first referral and treatment also appeared similar in all socio-economic and rural groups. Adjusting for disease stage and other variables, times to lung cancer treatment remained similar, but colorectal cancer treatment was quicker for outlying patients (adjusted hazard ratio 1.30; 95% confidence intervals 1.03 to 1.64). These findings suggest that socio-economic status and rurality may have a minor impact on modalities of treatment for colorectal and lung cancer, but do not lead to delays between referral and treatment.

*British Journal of Cancer* (2002) **21**, 585–590. doi:10.1038/sj.bjc.6600515 www.bjcancer.com

© 2002 Cancer Research UK

## 

Lung and colorectal cancers are two of the most commonly diagnosed cancers and the most common causes of cancer related death in Scotland (ISD, 2001). Several studies in different countries have found that survival from these cancers varies with socio-economic and geographical factors (Kogevinas and Porta, 1997; Campbell *et al*, 2000). In a recent analysis of Scottish cancer registry data, survival from lung and colorectal cancer was poorer for patients resident in the most deprived areas compared to those in the least deprived areas (Mclaren and Bain, 1998). Another analysis of this data found that, compared with those living in towns and cities with cancer centres, adjusted survival for patients living in rural areas was 9% poorer for lung and 11% poorer for colorectal cancer (Campbell *et al*, 2000).

Stage at diagnosis and treatment are the principal determinants of cancer survival (Auvinen and Karjalainen, 1997). We and others have shown that patients in rural areas have more advanced disease at diagnosis (Liff *et al*, 1991; Launoy *et al*, 1992; Campbell *et al*, 2001) but the relationship between stage and socio-economic status remains unclear with conflicting results in different studies (Auvinen and Karjalainen, 1997; Ionescu *et al*, 1998). With regard to treatment, there are some indications that management is poorer for rural patients with lung cancer in North America (Greenberg *et al*, 1988) and colorectal cancer in France (Launoy *et al*, 1992). Similarly, deprived patients with colorectal cancer were found to have poorer treatment in Finland (Auvinen and Karjalainen, 1997). A study of computerised hospital discharge data in Scotland has suggested that patients with colorectal cancer from deprived areas are less likely to be treated with chemotherapy (Mcleod, 1999). Overall, however, research findings have been conflicting and little has been reported in the United Kingdom (Auvinen and Karjalainen, 1997). In this study, we investigated whether there were variations in treatment of colorectal and lung cancer with socio-economic deprivation and urban/rural residence.

## PATIENTS AND METHODS

This was a historical cohort study. Details of sampling and data collection have been described previously (Campbell *et al*, 2001). Briefly, all patients diagnosed with colorectal or lung cancer in north or northeast Scotland in 1995 and 1996 were identified by the Scottish cancer registry and a random sample of 1398 selected, weighted to ensure equal numbers of lung and colorectal cancers and urban and rural participants. Sets of case notes could be obtained from teaching and general hospitals in Grampian and Highland for 1323 (95%) of the cohort. Clinical data were abstracted in a standardised manner. There were no important differences in patient characteristics between cases whose notes were reviewed and those whose notes were not traced (Campbell *et al*, 2001). Nine patients died the same day that they were diagnosed so were excluded from follow up, leaving 1314 cases for analysis.

The main outcomes of the study were surgery, radiotherapy and chemotherapy within 1 year of diagnosis and the length of time between first referral (date of referral letter, or first contact with hospital if there was no referral) and first treatment with surgery, radiotherapy or chemotherapy.

The main independent variables were material deprivation (as a proxy for socio-economic status) and urban-rural status. Indicators of deprivation and urban-rural status were assigned to cases according to their ‘output area’ of residence. Output areas, which are the smallest unit of population on which census data are available in Scotland (median population 130), are more sensitive than larger areas when measuring socio-economic deprivation and geographical location in rural areas (Reading *et al*, 1993; Campbell *et al*, 2000). Carstairs deprivation scores were calculated from 1991 census data at the output area level and grouped into population quintiles (Carstairs and Morris, 1990). Distance to the nearest cancer centre (in Aberdeen or Inverness) was used as the basis of the indicator of urban-rural status because it has been found to be associated with poorer survival in previous research in Scotland (Campbell *et al*, 2000). Patients were assigned to one of four predefined categories: 0 to 5 km, 6 to 37 km, 38 to 57 km and ⩾58 km (Campbell *et al*, 2001). Other variables considered in the analysis were sex, age, settlement size, health board of residence, previous history of cancer, and presentation (emergency hospital admission or not). Cancer site (colon or rectum) and Dukes stage were considered in the analysis of colorectal cancer, and tumour histology (non-small cell or small cell) and ISS stage in the analysis of lung cancer.

Data were managed using Microsoft Access and analysed using SPSS for Windows release 9. Data on the two cancers were analysed separately. Proportions of cases receiving surgery, radiotherapy and chemotherapy were compared using the chi-square test and modelled using logistic regression. Differences in time between referral and first treatment were compared using Kaplan–Meier curves and the log rank test and modelled using Cox regression.

## RESULTS

In all, 661 cases with lung cancer and 653 with colorectal cancer were included in the analysis. Selected characteristics are shown in Table 1

**Table 1:**
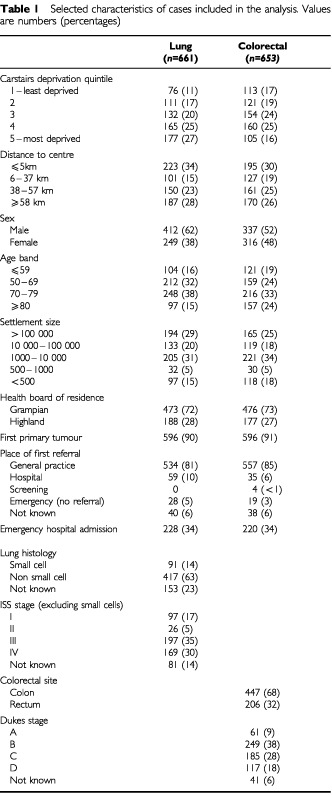
Selected characteristics of cases included in the analysis. Values are numbers (percentages)

. For both types of cancer, more than 80% of patients were referred from general practice and a third were emergency hospital admissions.

Of 653 patients with lung cancer, 85 (13%) had surgery, 412 (63%) radiotherapy and 124 (19%) chemotherapy in the first year after diagnosis (details of treatment were incomplete for eight patients). For colorectal cancer, 583 out of 642 (91%) patients had surgery, 145 out of 642 (23%) chemotherapy and 82 out of 643 (13%) radiotherapy (data on surgery and chemotherapy were incomplete for 11 patients and on radiotherapy for 10 patients). On univariate analysis, there were few differences in proportions of patients receiving surgery, chemotherapy and radiotherapy by either deprivation or rurality (Table 2

**Table 2:**
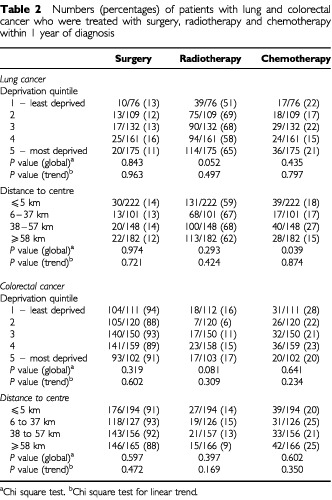
Numbers (percentages) of patients with lung and colorectal cancer who were treated with surgery, radiotherapy and chemotherapy within 1 year of diagnosis

). Of the potential confounding variables, disease stage was strongly associated with the likelihood of all three forms of treatment for both cancers. Age, health board of residence, mode of presentation (emergency admission or otherwise), and cancer site (colon or rectum) were associated with some treatments. Table 3

**Table 3:**
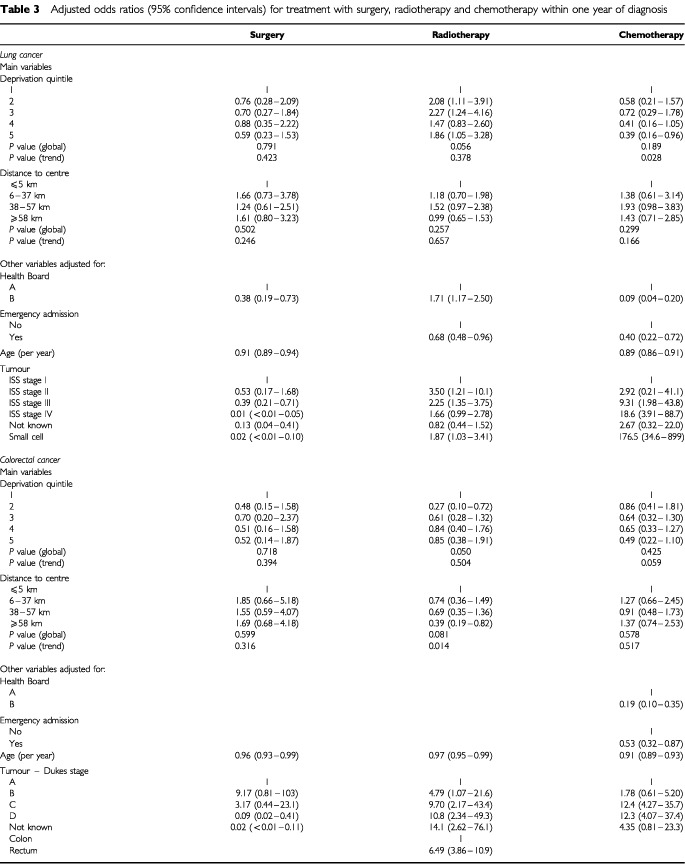
Adjusted odds ratios (95% confidence intervals) for treatment with surgery, radiotherapy and chemotherapy within one year of diagnosis

shows the adjusted odds ratios for treatment with surgery, radiotherapy and chemotherapy taking account of these variables. The adjusted figures suggest that chemotherapy for both cancers was less likely with increasing deprivation and these trends are shown in Figure 1

**Figure 1:**
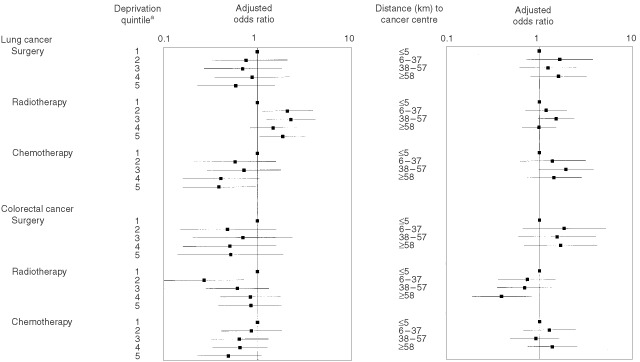
Adjust odds ratios (95% confidence intervals) for treatment with surgery, radiotherapy and chemotherapy within 1 year of diagnosis. ^a^Least deprived is ‘1’ and most deprived ‘5′.

. The trend was borderline insignificant for colorectal cancer (*P*=0.059), but significant for lung cancer (*P*=0.028) where the odds ratio for chemotherapy in the most deprived group compared to the most affluent was 0.39 (95% confidence interval 0.16 to 0.96). With regard to rurality, the likelihood of radiotherapy for colorectal cancer decreased with increasing distance from the cancer centre (*P* (trend)=0.014). The odds ratio for outlying patients with colorectal cancer compared to those resident closest to the centre was 0.39 (95% CI 0.19 to 0.82).

Overall, the median time between first referral from any source and first treatment with surgery, radiotherapy or chemotherapy was 34 days for lung cancer and 37 for colorectal cancer. For lung cancer, there were no differences with deprivation or rurality in either univariate or multivariate (taking account of stage, age, and health board) analyses (Table 4

**Table 4:**
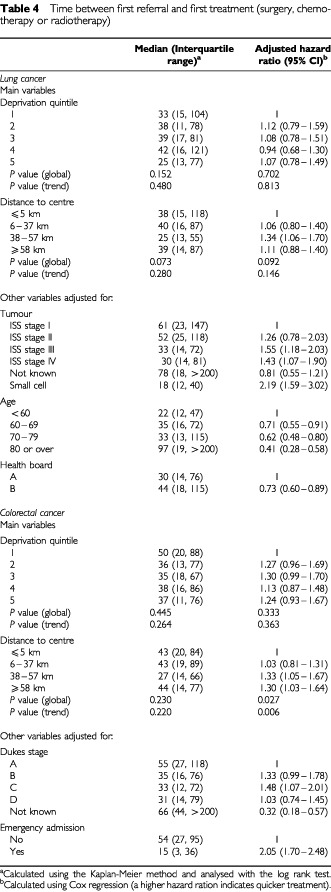
Time between first referral and first treatment (surgery, chemotherapy or radiotherapy)

and Figure 2

**Figure 2:**
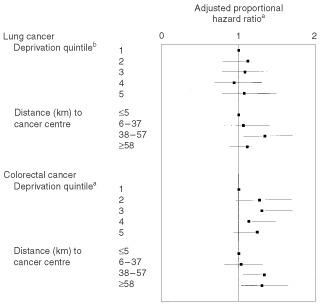
Adjusted proportional hazard rations for time between first referral and first treatment (surgery, chemotherapy or radiotherapy). ^a^A higher proportional hazard ration indicates faster treatment. ^b^Least deprived is ‘1’ and most deprived ‘5′.

). For colorectal cancer, there were again no differences on univariate testing, but adjusting for other significant variables (stage and emergency admission to hospital), outlying patients were treated more quickly (Table 4 and Figure 2). The hazard ratio for treatment in patients living more than 58 km from a cancer centre compared to those within 5 km was 1.30; 95% confidence interval 1.03 to 1.64.

## DISCUSSION

We found that in the north and northeast of Scotland, there was limited evidence that deprivation and rurality were associated with differences in treatment. There may be some impact on treatment modalities, but no worsening of treatment delay.

This study has a number of strengths and limitations. The Scottish Cancer Registry has high levels of case ascertainment over a long period, being reported as at least 96% complete (Brewster *et al*, 1997). In this study, the rate of case note retrieval (95%) was high and we have previously shown that there were no important differences between cases whose notes were retrieved and those whose notes were not (Campbell *et al*, 2001). The setting for the study had two cancer centres located in the two main cities, reasonably close to about half their populations, but with the remainder spread over a large rural area. This made the comparison of rural and urban areas relatively straightforward. On the other hand, comparison of deprivation categories was more difficult. North and Northeast Scotland do not have the same high levels of deprivation seen in some other areas (for example, the central industrial belt of Scotland)–there are, however, significant pockets of deprivation and overall the area is less affluent than, for example, England and Wales (Carstairs and Morris, 1990). A second problem is that levels of deprivation are more difficult to assess in rural areas where affluence and poverty can coexist in close proximity. In an attempt to improve sensitivity, we calculated deprivation scores at the level of the smallest area possible–this method has been found sensitive enough to detect survival differences for common cancers in Scotland (Campbell *et al*, 2000). We were aware of the importance of disease stage in determining subsequent treatment and would have liked to have presented stage specific analyses, but our numbers were not large enough for these to provide meaningful findings. We have, instead, adjusted for stage at diagnosis in our analyses. Finally, the data we collected was limited to that which we could readily and reliably obtain from case notes. We were not, for example, able to collect data on WHO performance status or multidisciplinary team meetings. This meant that, although we were able to compare mode and speed of treatment, we were not able to investigate quality of treatment within each modality. We cannot, therefore, exclude important differences in the quality of treatment to patients in the groups studied, although the fact that all patients (rural and urban, affluent and deprived) in each health board area received specialist oncology from one hospital, suggests that this is unlikely.

Our findings add to limited evidence on whether differences in treatment contribute to poor survival among socio-economically deprived people with lung and colorectal cancer (Auvinen and Karjalainen, 1997). In a previous analysis of this dataset, we found no evidence of more advanced stage at diagnosis among deprived patients (Campbell *et al*, 2001). In the current study, although times between referral and treatment appeared to be long in many cases (particularly compared to the recommendations published in the NHS Cancer Plan (Department of Health, 2000)), they were equally long in all deprivation categories. We found lower likelihood of deprived patients receiving chemotherapy, but this trend was only detected after adjusting for other variables (and not on univariate analysis), so needs to be treated with an element of caution. On the other hand, the trend was present for both cancers and is in line with a previous study of colorectal cancer treatment in Scotland, which found the odds ratio of chemotherapy to be 0.73 in deprived compared to affluent areas (Mcleod, 1999). Lower rates of chemotherapy may be a contributory factor in the poor survival rates of socio-economically deprived people with lung and colorectal cancer in Scotland (Mclaren and Bain, 1998), but confirmation in further research would be helpful. Others have reported high rates of co-morbidity among deprived patients and suggested that this may explain their survival disadvantage (Macleod *et al*, 2000). It could also explain a tendency for less use of chemotherapy.

With regard to patients in rural areas, we have previously reported that they have more advanced disease at diagnosis (Campbell *et al*, 2001). In a qualitative study, they expressed concern that their route from referral to diagnosis and treatment was more complicated (involving peripheral hospitals and outreach clinics) and therefore slower (Bain and Campbell, 2000). In this study, however, we found no evidence of increased delays between referral and treatment–in fact, treatment appeared to be quicker for patients from outlying areas after adjusting for disease stage and emergency admissions. The only difference in treatment we detected was less radiotherapy for colorectal cancer. This finding is in line with research in the United States which suggested that travelling distance was taken into account when considering treatment options of uncertain benefit (Greenberg *et al*, 1988). Radiotherapy was not a standard treatment in Scotland for the majority of patients with colorectal cancer at the time patients in this study were diagnosed (1995–1996) (SIGN, 1997). On the other hand, where radiotherapy was a standard treatment (in lung cancer (SIGN, 1998)), there were no differences in treatment rates. We have previously reported that more advanced disease at diagnosis in rural patients is probably the main reason for their poorer survival (Campbell *et al*, 2001). Our current findings suggest that they are not substantially disadvantaged after diagnosis in terms of the treatment they receive, and that any delays in diagnosis occur before the point of referral.

In conclusion, deprivation and rural factors may have some impact on treatment of colorectal and lung cancer. It seems unlikely, however, that they are the most important factors contributing to inequalities in survival, at least in Scotland. For patients in outlying areas, more advanced stage at diagnosis remains the most important factor. For the socio-economically deprived, the reasons for poor survival remain unclear, but are likely to involve more complex factors than stage at diagnosis and treatment.

## ACKNOWLEDGEMENTS

This study was funded by a Cancer Research UK Primary Care Oncology Fellowship. Thanks to all at the Scottish Cancer Registry (at the Information and Statistics Division for NHS Scotland) and in particular, Veronica Harris, who extracted the registry data we sought. We would also like to thank the medical records departments at hospitals throughout north and northeast Scotland for their help finding case notes.
